# Bilateral concomitant inguinal and femoral hernias in an adult male: A case report

**DOI:** 10.1016/j.ijscr.2022.107504

**Published:** 2022-08-13

**Authors:** Franck Katembo Sikakulya, Sonye Magugu Kiyaka, William Mauricio Lopez Andino, Ahmed Kiswezi

**Affiliations:** a Faculty of Clinical Medicine and Dentistry, Department of Surgery, Kampala International University Western Campus, Ishaka-Bushenyi, Uganda; b Faculty of Medicine, Université Catholique du Graben, Butembo, Democratic Republic of the Congo

**Keywords:** Bilateral, Inguinal, Femoral, Hernias, Concomitant

## Abstract

**Introduction and importance:**

Bilateral concomitant inguinal and femoral hernias are an uncommon presentation clinically. If not managed in time, femoral hernias may be associated with incarceration or even strangulation.

**Case presentation:**

We report the case of a 75-year-old male who presented with bilateral inguinal and femoral swellings which were diagnosed clinically and by ultrasound scan as uncomplicated inguinal and femoral hernias. Surgical management was done using the inguinal approach. His postoperative period was uneventful and was discharged without any complaint on the 4th post-operative day.

**Clinical discussion:**

Bilateral concomitant inguinal and femoral hernias are a rare presentation. Surgery was done as the best option to prevent complications. The literature suggests that it remains the gold standard of management in groin hernias in adults.

**Conclusion:**

We present a rare condition in a 75-year-old male with uncomplicated bilateral concomitant inguinal and femoral hernias.

## Highlights

• We reported a first African case of bilateral inguinal and femoral hernias in a 75-year-old male who underwent Lotheissen and McVay operation.
• Patient presented with 2 left and right groin swellings confirmed clinically and by ultrasound scan as uncomplicated inguinal and femoral hernias.
• Surgery remains the management of choice for groin hernias.

## 1 Introduction

Although inguinal hernias constitute 75 % of abdominal wall hernias, in adults 2–8 % are femoral hernias, which often occur in older men [1], [2]. Femoral hernias are more frequently associated with complications such as incarceration and strangulation, which can lead to significant mortality and morbidity if not managed in time [3]. The simultaneous appearance of more than two bilateral inguinal and femoral hernias is a very unusual [4]. We report the presentation and surgical management of a 75-year-old male with concomitant bilateral uncomplicated inguinal and femoral hernias, according to SCARE 2020 criteria [5].

## 2 Case presentation

**Fig. 1:**
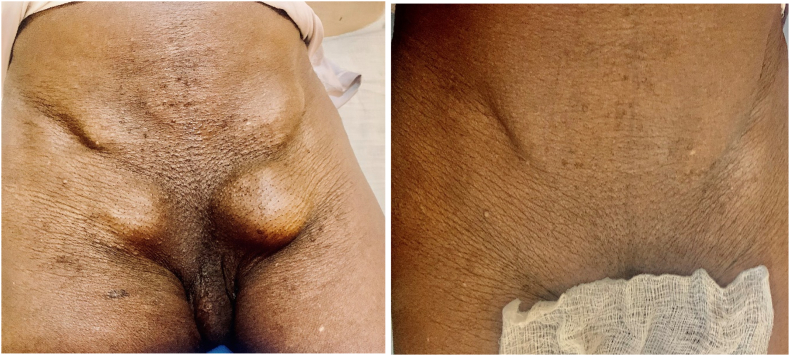
Clinical photograph showing preoperative bilateral inguinal and femoral hernias.

**Fig. 2:**
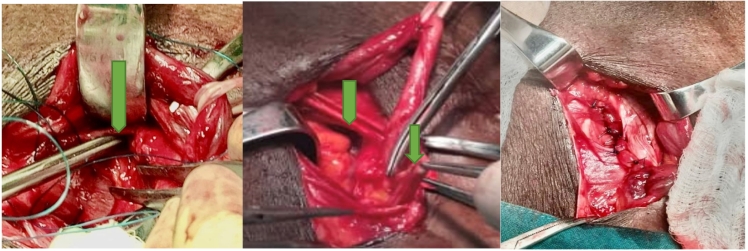
Clinical photograph showing intra-operative view of inguinal and femoral hernias (green arrows). (For interpretation of the references to color in this figure legend, the reader is referred to the web version of this article.)

**Fig. 3:**
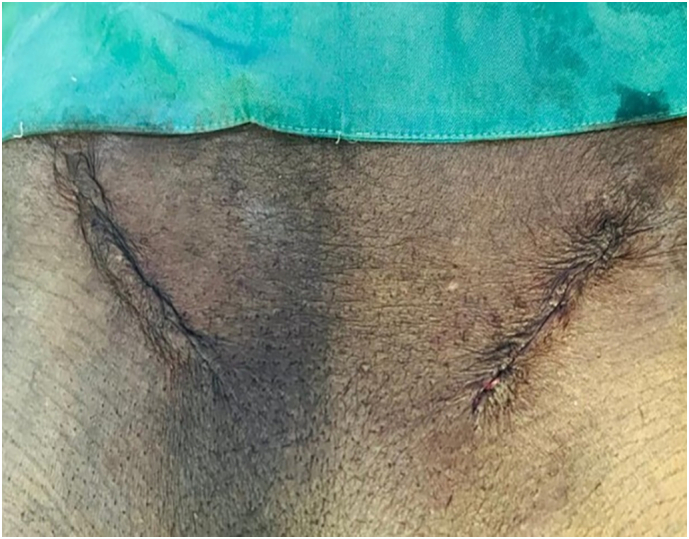
Clinical photograph showing post-operative view of bilateral inguinal and femoral hernias.

A 75-year-old male farmer consulted our teaching hospital with a history of bilateral painless inguinal and femoral swellings for five years. The swellings increased in size when coughing or while carrying heavy luggage. However, it was not associated with constipation or vomiting. The inguinal swellings were ovoid and measured 3 × 3 cm on the left and 2 × 2 cm on the right, and the femoral swellings were 3 × 4 cm on the left and 2 × 3 cm on the right [Fig. 1]. All four swellings had positive cough impulses, were reducible and non-tender and an ultrasound scan confirmed them to be bilateral inguinal and femoral hernias but no comments were made on the contents of the sacs. There was no history of previous surgery or any chronic disease such as chronic obstructive airways disease, constipation, or of low urinary tract symptoms. Routine pre-operative hematological investigations were all within the normal range. The patient did not have history of allergy to local anesthesia and consented for elective bilateral herniorrhaphies under spinal anesthesia. Surgery was carried out under spinal anesthesia, and normal fat tissues were the only contents of the sacs [Fig. 2]. The hernia contents were reduced into the peritoneal cavity and Lotheissen and McVay incisions were used to repair the defects. The patient received 2 g of ceftriaxone (50 mg/kg) 30 min prior to the surgery and pain in post-operative period was controlled by 75 mg diclofenac suppository twice daily for three days. The patient reported being satisfied with the technique used and was discharged on day three post-surgery [Fig. 3]. On the 14th post-operative day, he was reviewed and reported no complaints.

## 3 Discussion

A hernia is a protrusion of abdominal a viscus or part of it from its containing cavity. A femoral hernia is the protrusion of a viscous through the femoral canal due to a defect in the femoral ring [6]. Inguinal hernia constitutes about 75 % of anterior abdominal wall hernias and femoral hernias are usually unilateral and accounts for 2–4 % of groin hernias and more prevalent in female than males [1], [7]. Our case provides a rare phenomenon of crural hernia occurring bilaterally with simultaneous bilateral inguinal hernia. To the best of our knowledge, this is the first to be documented in Africa.

Femoral hernia is more susceptible to strangulation than inguinal hernias and there are identifiable risk factors associated with groin hernia [8], [9]. Some identifiable risk factors can increase the occurrence of groin hernia and are divided into two general categories namely, patients related risk factors and external risk factors [10]. These factors include smoking, increased intraabdominal pressure, systemic connective tissue disorders, low body mass index, old age, and male gender [11]. In this case the patient was male with advanced age, and this was the underlying risk factors.

Generally inguinal and groin hernias present as a lump which may cause a dragging sensation. Painful lumps are usually due to incarceration and with obstruction or strangulation will present with colicky abdominal pain and vomiting [3], [12]. These groin masses should be differentiated from lipoma, saphena varix, enlarged femoral lymphadenopathy, femoral aneurysm, and psoas abscess [7]. In this case the patient presented with inguinal and crural swellings bilaterally which were non-tender and had a positive cough impulse.

Different imaging modalities are used to confirm the diagnosis of hernias, and they include herniorrhaphy, ultrasonography, computed tomography (CT) and magnetic resonance imaging (MRI) [8]. Ultrasonography in this case showed bilateral femoral and inguinal hernia with no comment on the sacs as this modality is user dependent [8].

Operative management is the mainstay of treatment to repair the defects as soon as possible. This can either be done laparoscopically or through open approaches. Open approaches include McVay (high), Lotheissen's (trans inguinal) and Lockwood (low) among others for crural hernias [11]. Laparoscopic approach is the gold standard technique for repair of these hernias because of decreased postoperative pain, good view of surgical field, easy observation, fewer infection, and quick return to daily activities [13]. Two approaches can be employed namely TEPP (Totally extraperitoneal approach) or TAPP (Transabdominal preperitoneal approach) [14]. In this case the open technique of Lotheissen and McVay was used to repair the hernias. The patient's post-operative recovery was uneventful, and he was discharged three days after the operation. His follow-up was uneventful 2 months post-surgery.

## 4 Conclusion

Bilateral femoral hernias with concurrent bilateral inguinal hernia are an uncommon occurrence and, therefore, there is no data on optimal management. In this case, although all four hernias had probably been present for at least five years without complications, we opted for elective surgical repair. This decision was based on the well documented risk of complications associated with femoral hernias. This is presumed to be the first reported case in Africa, and this highlight the importance of proper history taking and clinical examination to decrease morbidity and risk of mortality due to complications.

## Provenance and peer review

Not commissioned, externally peer-reviewed.

## Funding

There was no external funding source for this report.

## Ethical approval

Not applicable.

## Consent

Written informed consent was obtained from the patient for publication of this case report and accompanying images. A copy of the written consent is available for review by the Editor-in-Chief of this journal on request.

## Author contribution

FKS managed the patient and wrote the first draft. SMK, WMLA and AK helped in editing and reviewing the paper. All authors read and approved the final version to be published.

## Registration of research studies

Not applicable.

## Guarantor

Franck Katembo Sikakulya.

## Declaration of competing interest

The authors declare no conflicts of interest.

## References

[1] Jenkins J.T., O'Dwyer P.J. (2008). Inguinal hernias. BMJ.

[2] Tsuchiya Y., Momose H., Kure K. (2021). Case of incarcerated femoral hernia treated with laparoscopic surgery after groin hernia repair. Case Rep. Gastroenterol..

[3] Sucandy I., Kolff J.W. (2012). Incarcerated femoral hernia in men: incidence, diagnosis and surgical management. NAM J. Med. Sci..

[4] Yoong P., Du_y S., Marshall T.J. (2013). The inguinal and femoral canals: a practical step-by-step approach to accurate sonographic assessment. Indian J. Radiol. Imaging.

[5] Agha R.A., Franchi T., Sohrabi C., Mathew G., for the SCARE Group (2020). The SCARE 2020 guideline: updating consensus Surgical CAse REport (SCARE) guidelines. Int. J. Surg..

[6] Nikolopoulos I., Oderuth E., Ntakomyti E., Kald B. (2014).

[7] Matsevych O.Y., Koto M.Z., Becker J.H.R. (2016). CASE REPORT – OPEN ACCESS International Journal of Surgery Case Reports Multiple concurrent bilateral groin hernias in a single patient; a case report and a review of uncommon groin hernias: a possible source of persistent pain after successful repair. Int. J. Surg. Case Rep..

[8] Leite T.F., CAA Chagas, LAS Pires, Cisne R., Babinski M.A. (2016). De Garengeot's hernia in an 82-year-old man : a case report and clinical significance.

[9] Mahajan A., Luther A. (2014). Incarcerated femoral hernia in male: a rare case report.

[10] Ogbuanya A.U., Olisa F.U., Ewah R.L., Nweke O., Ugwu N.B. (2020). Femoral hernia: epidemiology and challenges of management in a sub-Saharan African Country.

[11] Torabi H., Shirini K., Ghaffari R. (2022). Simultaneous bilateral femoral, direct, and indirect inguinal hernia in a single patient: a casereport.

[12] Uesato Y. (2020). Case report a case of bilateral concomitant inguinal and femoral hernias treated with transabdominal preperitoneal repair.

[13] Kochupapy R.T., Ranganathan G., Dias S., Shanahan D., Hospital P.P. (2013). Aetiology of femoral hernias revisited : bilateral femoral hernia in a young male (two cases).

[14] Wong M., Javid P.J. (2020). Journal of pediatric surgery case reports bilateral femoral hernias in a male child as the initial presentation of Ehlers-Danlos syndrome. J. Pediatr. Surg. Case Rep..

